# A critical review on altmetrics: can we measure the social impact factor?

**DOI:** 10.1186/s13244-021-01033-2

**Published:** 2021-07-02

**Authors:** Cristina García-Villar

**Affiliations:** Radiology Department, Hospital Universitario Puerta del Mar, Ana de Viya Avenue, nº 21. 11009, Cádiz, Spain

**Keywords:** Altmetrics, Bibliometric indicators, Radiology, Medical imaging, Social media

## Abstract

Altmetrics measure the digital attention received by a research output. They allow us to gauge the immediate social impact of an article by taking real-time measurements of how it circulates in the Internet. While there are several companies offering attention scores, the most extensive are Altmetric.com (Altmetric Attention Score—AAS) and Plum X (Plum Print). As this is an emerging topic, many medical specialities have tried to establish if there is a relationship between an article’s altmetric data and the citations it subsequently receives. The results have varied depending on the research field. In radiology, the social network most used is Twitter and the subspeciality with the highest AAS is neuroimaging. This article will review the process involved from the start when an article is published through to finally obtaining its altmetric score. It will also address the relationship between altmetrics and more traditional approaches focusing on citations in radiology and will discuss the advantages and limitations of these new impact indicators.

## Key points

- Altmetrics measure the digital attention received by an article using multiple online sources.
- Altmetrics should not been seen as alternatives, but rather complementary, to more traditional measurements.
- In radiology, articles with nonimaging content (for example, education, quality or safety) have more altmetric data that those with more radiology-specific content. Within subspecialities, articles on neuroimaging are those that gain most attention.

## Background

Bibliometric indicators (BI) are numerical data linked to the production and consumption of scientific works [1]. The BI are calculated objectively using a large volume of data available in international reference databases [2]. As research results are shared through publications, the BI have traditionally evaluated scientific production and its impact on the community [3]. In general, the BI can be classified by whether they apply to authors or research groups (for example, the H index or collaboration indices) or if they are specific to journals (for example, the Impact Factor, the Eigenfactor or the Scimago Journal Rank) [4].

The Internet’s evolution over recent years has enabled the creation of academic social networks and this has, in turn, brought about significant changes to the way science is disseminated [5]. This new way of distributing information is possible thanks to the development of “Web 2.0” or the “Social Web” which enables its users to interact and collaborate, thus making contributions to, sharing and commenting on content [6].

Given the social and communicative nature of science, many researchers have started using social networks, blogs, repositories (virtual spaces where articles are stored and accessed to download) and other platforms which can be used to share and access scientific information [6]. In this context emerges the term, altmetrics, to define some new indicators that analyse the social impact and visibility of a scientific publication [7–9].

This article aims to help the reader interpret the altmetrics, understand their relationship with traditional citations and discuss their advantages and limitations.

## Sources, providers and attention scores

**Fig. 1:**
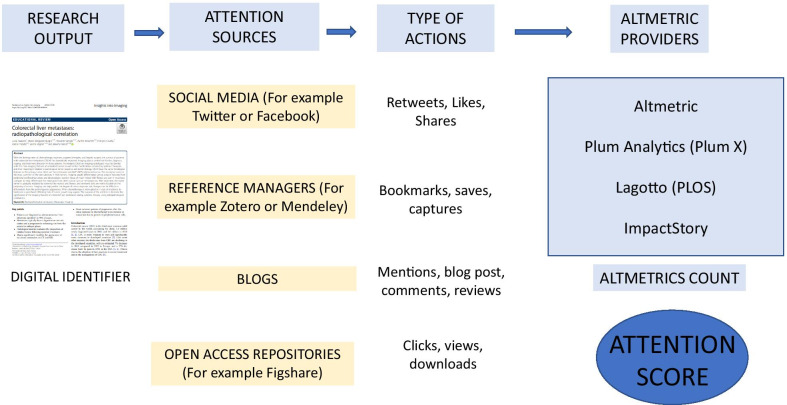
Process used to obtain ATTENTION SCORE. Starts with the publication of a research output which has an assigned Digital Identifier. Different actions are performed on the different attention sources (for example, saves, captures, mentions, etc.) These are all integrated by the altmetric provider (the most extensive are Altmetric.com and Plum Analytics) which after applying their own formulas, determine the Attention Score (Altmetric Attention Score in the case of Altmetric and Plum Print in Plum Analytics)

The process which starts with the research output’s publication through to obtaining its attention score is complex and meticulous. Figure 1 is a summary of all the stages involved and is explained in more detail below.

## Research output

It is possible to obtain an Attention Score for any scientific work (research output) (books, book chapters, academic articles, presentations, theses, grey literature, clinical trials, etc.). The only thing required to be able to record the online attention of any given document is that it has at least one digital identifier (DI).

The type of digital identifier depends on the publication type [7]. For example, academic articles are identified by the Digital Object Identifier (DOI) or PubMed Identifier (PMID), a book by its International Standard Book Number (ISBN) and a clinical trial by its ClinicalTrials.gov Identifier (NCT Number) [10].

Depending on the repository, the research output may also be identified with a different ID. For example, the arXiv repository which indexes articles from mathematics, physics and quantitative biology uses arXiv IDs [11]; or the Social Science Research Network (SSRN) IDs associated with the SSRN, owned by Elsevier, which incorporates articles from various disciplines such as Health Science and Social Science and Humanities [12].

There are also researcher IDs, for example, the Open Research and Contributor Identifier (ORCID) [13].

Only links which contain the work's ID are included in the altmetric calculations [6]. This detail is important to note as it is common to share an image with the title and article authors but although this is a means of dissemination, it does not count for the altmetric calculation.

### Attention sources

Altmetrics measure the digital attention that an article receives by using data from different online resources. While sources vary widely, we can group the main ones into four main categories [5, 7, 14–16].

- Social Networks. Social networks, both general ones (such as Twitter [17], Facebook [18] or YouTube [19]) and scholarly social networks (SlideShare [20], LinkedIn [21], ResearchGate [22] or Academia.edu [23] can be used by researchers to disseminate publications and network with other professionals [24].
- Online reference managers or Bookmarking sites. These allow users to save or insert citations online and share this information with other professionals [25]. Potential interest is determined by the number of users that save any given article. The most well known are Zotero [26] and Mendeley [27]
- Blogs: In order to calculate the altmetrics, all blog types, both science blogs and more general ones, are included. For a blog to be classified as a science blog (for example, Researchblogging website [28]), it has to not only deal with scientific subject matter, but also be produced by esteemed members of the scientific and academic community (a researcher, university professor, scholarship researcher or even a science journalist) [29]. General blogs can be published by anyone.
- Open Access repository: These are digital platforms for academic research which can be accessed immediately, permanently and free of charge. Examples include Figshare [30] and Directory of Open Access Journals (DOAJ) at Lund University [31].

In addition to these four groups, there are also many other sources which act as altmetric inputs [32] including Faculty of 1000 (F1000) [33] which brings together and evaluates the most important published articles in any given field (including medicine) following the recommendations given by a team of scientists; collaborative encyclopaedias such as Wikipedia [34]; policy documents and social news websites such as Reddit [35].

### Type of actions

Published work is disseminated and professionals receive it without actively seeking. If it looks interesting to us, we may adopt an active role and share it, download it or comment on it thus contributing to its increase in visibility and dissemination [8]. This affects the article’s social impact.

Each source has an associated action of one type or another [9, 36–38]:

- Likes and shares: Social Networks
- Viewed: HTML views, PDF downloads
- Discussed or Mentions: blog post, comments, reviews, policy documents, Wikipedia.
- Saved or Captures: bookmarks and saves in electronic Reference Managers.

Plum X [39] includes a fifth action, the citations. This one combines traditional citations received from citation indices such as Scopus or PubMed Central) and new citations such as clinical citations (Clinical Guidelines), policy citations or patent citations which help evaluate the social impact.

### Altmetric providers

There are several companies which offer altmetric services. Each one tracks a combination of different sources (even though many coincide) and uses different formulas to calculate the attention score [40]. Table 1 summarises the main sources used by each company. The most extensive are Altmetric and Plum X.

**Table 1:** Comparison of the most representative altmetric providers

	Altmetric.com	PlumX	Impactstory	Article-level metrics-PLoS
Created time	2011	2012	2011	2009
Target group	Researchers, publishers, librarians, editors, funders	Researchers, publishers, funders	Researchers, publishers, funders	Researchers, publishers, funders
Data source	Mainstream media (This list currently extends to over 5000 English and non-English global news outlets) Blogs (15.000 academic and no academic blogs) Wikipedia pages Policy Documents Patent citations Social networks (Twitter, Facebook, Reddit) Post-publication peer review fórums (Publons/Pubpeer) References Manager (CiteULike) Other online sources: Sites running Stack Exchange (Q&A) F1000Prime recommendations YouTube Open Syllabus Citations (Dimensions and Web of Science) Citations are only available within the Explorer	Scopus, CrossRef, PubMed Central EBSCO, PLOS, bit.ly, GitHub, Dryad, Figshare, SlideShare, Institutional Repositories, WorldCat CiteULike, Mendeley, Delicious, SlideShare, YouTube, GitHub, Goodreads, Vimeo Blog posts, comments, reviews, Wikipedia references, news media) Social Media: Facebook, Reddit, SlideShare, Vimeo, YouTube, GitHub, StackExchange, Wikipedia, SourceForge, Research Blogging, Science Seeker, Amazon, Google Plus, Twitter via DataSift)	Scopus, Web of Knowledge, HighWire, Google Scholar Citations, PubMed CiteULike, Mendeley, CrossRef, Vimeo, Figshare, GitHub, SlideShare, YouTube, Delicious Social Media (Twitter, Facebook, Blogs, Figshare, Wikipedia, Vimeo, YouTube, SlideShare, Delicious, GitHub)	(PLOS Journals, PubMed Central) CiteULike, Mendeley CrossRef, DataCite, Europe PMC, PubMed Central, Scopus, Web of Science F2000 Prime PLOS Comments, Facebook, Reddit, Twitter, Wikipedia
Type of actions	Social media Viewed Discussed or Mentions Saved or captures	Citation Usage Captures Mentions Social media	Citation Captures Social Media	Viewed Saved Cited Recommended Discussed
Attention Score	Altmetric Attention Score	Plum Print		
Accessibility	Annual subscription basis	Particular institutions	Free Access	Free access
Coverage	Scholarly articles	Journal articles, videos, books, presentations…	All the research products (Journal articles, blog posts, dataset…)	Papers from PLOS
Business model	For profit	For profit	Non-profit	Non-profit

These platforms are key research tools with regard to altmetrics and are increasingly used to evaluate articles, authors and research [41].

#### Altmetric

Altmetric (www.altmetric.com) [42] was created in 2011 by Euan Adie with funding from Digital Science. It's important to differentiate the term altmetrics (the general term used to define these new “social impact” indicators) from “Altmetric” or “Altmetric Attention Score” (AAS) which are specific to this company [42].

This company is used by publishers such as Springer, Nature, Publishing Group and Biomed Central [9] and is included in repositories such as the University of Queensland institutional repository.

The altmetric score for this company is referred to as the Altmetric Attention Score (AAS).

Three factors influence the altmetric calculations [42]:

- Volume (how many times an article is mentioned)
- Sources (where do the mentions come from)
- Authors (of each mention, in order to not count the times an author interacts with his/her own work)

The AAS total is a number that is calculated depending on the source and frequency that it has been used [5, 9]. For example, a mention in a blog has a higher value than a Tweet (Table 2). Exactly how it is calculated is not known making it impossible for an individual to calculate the index [43].

**Table 2:** Sources and their weights in calculating AAS (Dates from Altmetric.com)

Sources	Weight
News	8
Blogs	5
Wikipedia pages; Policy Documents; Patents	3
Twitter (tweets and retweets); Peer review (Publons, Pubpeer); Weibo (until 2015); Google + (until 2019); F1000; Syllabi (Open Syllabus)	1
LinkedIn (until 2014)	0.5
Facebook; Reddit; Pinterest (until 2013); Q and A; YouTube	0.25
Mendeley/Web Science citations	0

#### Plum X

This was created in 2012 by Andrea Michalek and Michael Buschman from Plum Analytics [39, 44, 45]. In 2017, Plum Analytics was purchased by Elsevier and so Plum X can track the online activity of any given article indexed in the Scopus database.

Plum X divides the sources into 5 categories [9, 44, 45], each represented by a different colour: usage (green); captures (purple); social media (blue); mentions (yellow) and citations (red). Plum Print is the graphic display of data used by Plum X. It does not provide a total score but rather indicates the number of metrics for each of the five categories, making it easier to understand than the previous example.

#### Lagotto (PLOS Article-Level Metrics)

Lagotto was the first altmetrics data provider, created in 2009 by the Public Library of Science (PLOS) [45]. It has only been adopted by three publishers (PLOS, Copernicus and Public Knowledge Project) [40].

#### ImpactStory

ImpactStory was developed by Jason Priem and Heather Piwowar in 2011 and can currently be found integrated in the Our Research website [46]. Unlike those already mentioned, this one builds the altmetric profile of a researcher rather than a piece of research. To do this, they use ORCID and a Twitter account which creates a profile with a list of their publications mentioned online [40]. The users create their curriculum vitae (CV) by uploading all their work (articles, slide presentations, posters, etc.). Following this, ImpactStory tracks where each item has been cited (using Scopus), where it has been seen and read (Mendeley) and how it has been discussed (Tweets and blog comments). For each item, it indicates how to cite it, its DOI and its PubMed ID. It also allows you to download the CVs [9].

#### Crossref even data

Crossref even data (CED) [47] was created in 2016. It is a more limited tool as, for each event, it only sets out the information linked to the DOI. For example, it shows an article's mention on Twitter but not the number of tweets [40].

## Altmetrics are not alternative metrics

Many studies have researched if there is a relationship between altmetrics and the number of citations an article goes on to receive [48], with Twitter being the most studied source.

Several authors [38, 49–54] have found a relationship between the number of tweets and the number of citations an article goes on to receive, although generally speaking, this correlation is low. Robinson-García [38] found that only 19% of the articles indexed on the Web of Science (WOS) had available altmetric data while Haustein [54] concluded that less than 10% of the articles indexed on PubMed are mentioned on Twitter. There may be a correlation between the number of tweets an article receives in the first 3 days and the number of citations it goes on to receive [50], but this assertion cannot be guaranteed as only one study has investigated this.

Many medical specialities have also analysed the relationship between the AAS (obtained from altmetric.com), the number of citations an article receives and the IF of the journal where it is published [55–64]. Even though the results vary significantly between the different specialities, generally speaking this correlation is also weak.

However, altmetrics appear to be constantly evolving and expanding. Some studies have analysed two time periods [55–60] revealing that there is a stronger correlation between the AAS, number of citations and IF for more recent articles.

Different factors can influence the level of social attention an article receives (and in turn, the Attention Score it achieves) and these include both the type of article and its topic: Editorials are the most popular type of articles on Twitter (yet they receive less citations) as opposed to more lengthy articles (which are shared less on social networks but are cited more often) [65]. Another observation is that more popular topics such as erectile dysfunction or sexual medicine have a higher Attention Score than other articles in urology even though they are not the ones most cited [66]. These aspects support the idea that altmetrics measure the social impact more than the academic or scientific impact which are measured by the BI.

Another line of investigation has researched the correlation between the number of citations and downloads on Mendeley (finding a moderate correlation in Nature, Science [67] and PLoS [68]) and between citations and the appearance of the articles in blogs. In these cases, the articles most discussed are the ones that went on to receive the most citations [69].

A higher or lower correlation with the number of citations depends on the provider. Peters [70] compared data from Plum X, ImpactStory and Altmetric.com with the number of citations, concluding that although the correlation was generally low, Plum X was the provider that showed the highest correlation.

Therefore, as most of the studies establish a low correlation between the AAS and the number of citations the article goes on to receive, generally speaking, altmetrics cannot be currently considered to be alternative metrics to the traditional BI as what they measure is the study’s social impact [9]. Popular topics and opinion articles usually have higher attention scores than an original article on a specific topic.

## Altmetrics and radiology

Even though Twitter is the social network most used by radiologists, the social network’s following is low [71]. Only 14 of the 50 journals with the highest impact factors have a Twitter profile [72]. The use of Instagram is limited to disseminating clinical cases and, in addition, is not included in the Attention Score calculations [73].

Blogs and citations have also been studied with regard to radiology, demonstrating that sharing scientific material on a blog promoted on social networks significantly increases the study’s dissemination [74].

At present, there are few original studies in diagnostic imaging that research altmetric dynamics.

Rosenkrantz [75] analysed original articles published in Academic Radiology, American Journal of Roentgenology, Journal of the American College of Radiology and Radiology, comparing the AAS and the number of citations received. Out of the 892 articles, almost all obtained at least one citation on WOS while only 41.8% had an AAS available. Mendeley, Twitter and Facebook were the sources most used. The most cited articles were those on topics strictly related to radiology while those dealing with topics that crossed into other fields (for example, educational aspects in radiology) were those that achieved the highest AAS. This resonates with the social aspect of altmetrics: the more cross-disciplinary the topic, the more interest it generates [4, 9].

Neuroimaging is the subspeciality with the highest AAS [76], and Frontiers in Human Neuroscience is the journal with the highest concentration of AAS followed by Radiology and Neurology [77].

Differences according to the type of article, the topic or publication date are also observed in nuclear medicine [78].

In image diagnostics, there are many subspecialities and topics for which there is no research on the workings of altmetrics or their relationship with the number of citations received. Nor have there been studies to investigate whether or not there exists a relationship between altmetrics and the publication date, as researched for other specialities. Therefore, there remains the need for more original studies in our field in order to arrive at more solid conclusions.

## Altmetrics in *Insights into Imaging*

In order to discover the characteristics of altmetrics in the journal *Insights into Imaging*, articles published from 1 July 2019 to 31 October 2020 were analysed. From the included articles’ web pages, the Altmetric.com browser plug-in was used to access the articles’ Altmetric Attention Score and detailed source information page. The overall score, as well as the number of mentions the article received on all listed online platforms, was also registered.

Of the 168 articles, 119 (71%) had some altmetric data registered and therefore had an AAS assigned (range 112–1). This data is superior to that obtained by Rosenkrantz [70] whose research, as previously discussed, revealed that less than half of the articles he analysed had some type of altmetric data. This reconfirms the exponential growth of altmetrics in recent years. Like other specialities, the source most used was Twitter (112 articles received at least one Tweet) followed by Facebook (49), blogs (4), news outlets (3) and Wikipedia (1).

The articles with the highest AAS did not feature a dominant topic but it is true that articles on artificial intelligence sparked most interest. It is also true that articles with the highest AAS were more cross-disciplinary as observed by Rosenkrantz [75] (Table 3).

**Table 3:** The ten articles with highest AAS published between July 2019 and October 2020 in *Insights into Imaging*

Title	Date	AAS	Mendeley	Twitter	Facebook	News outlet	Blog	Wikipedia
Chest imaging using signs, symbols, and naturalistic images: a practical guide for radiologists and non-radiologists	Dic 2019	112	87	144	6			
Deep learning workflow in radiology: a primer	Feb 2020	45	93	75				
Ethics of artificial intelligence in radiology: summary of the joint European and North American multisociety statement	Sept 2019	44	64	44	5	3		
Gender discrepancy in research activities during radiology residency	Dic 2019	41	16	103	1			
Sports-related lower limb muscle injuries: pattern recognition approach and MRI review	Oct 2020	39	9	98	1			
Structured report data can be used to develop deep learning algorithms: a proof of concept in ankle radiographs	Sept 2019	36	38	46	1		1	
Mentorship in academic radiology: why it matters	Nov 2019	32	11	48	2			
Imaging of skull vault tumors in adults	Feb 2020	31	20	55				1
Magnetic resonance imaging of the papillary muscles of the left ventricle: normal anatomy, variants, and abnormalities	Ag 2019	28	36	61	1			
Assessment of hepatocellular carcinoma treatment response with LI-RADS: a pictorial review	Dic 2019	27	26	43	1			

Date of search 13-11-2020

*AAS* Altmetric Attention Score

## The emerging role of altmetrics and their main limitations

Altmetrics enable us to view the immediate social impact an article has [79] thanks to a measurement which is provided in real time showing how an article is shared on the Internet [39]. They also provide immediate feedback to the researcher on the social interest that their work has generated [5, 7, 39]. This immediacy contrasts with the traditional BI which add up the citations an article receives over a period of 2 or 3 years [47, 80].

The growing increase in scientific publications makes it difficult for professionals to remain up-to-date. Both this and the fact that we are increasingly using social networks means that sharing information through the Internet has become the most common way that we keep ourselves informed. The information that we see when we look at our phones can constitute the first filter to know which articles are catching the attention of our colleagues [32].

But we have to interpret this scientific material sharing with caution: the fact that an article is shared or discussed on social networks does not guarantee it is of high quality [32]. When referring to general social networks (such as Twitter or Facebook) any user can share and help disseminate research without being an expert in the subject [47, 81]. In fact, as seen previously, the articles on erectile dysfunction are the ones with the highest AAS in urology and yet there is a weak correlation between this and the number of citations they go on to receive [66].

It should also be taken into consideration that the altmetrics are expressed in page visits or social network mentions. However, the act of sharing or downloading a study does not necessarily mean that the user has read and understood it or that it will be useful for the scientific community [79, 82]. Citing a scientific study involves reading the manuscript, critically analysing it, comparing the results and incorporating the citation in the article being drafted. All this work is far from the effort required to retweet a study [79]. Furthermore, traditional citations can be tracked, whereas, knowing who is behind shares is not always clear with altmetrics (you can act under a pseudo name or use several accounts at once) [37], meaning that altmetrics can be easily manipulated.

Some authors suggest that altmetrics are useful for assessing new researchers that have not had the opportunity to accumulate many citations in their short time in academia [79]. For this reason, some researchers have incorporated them into their curriculum vitae (CV) by asserting, for example, which articles have been recommended by Faculty of 1000 or considered “Highly Accessed” (in other words, a high number of downloads) or by incorporating data from AAS or Plum Print [42, 83]. However, this puts those authors who do not use social networks at a clear disadvantage [32, 37]. A researcher with 2000 followers on Twitter will have higher altmetric indices than one that does not use it.

Beyond their incorporation in CVs, there are authors who propose using altmetrics as criteria for distributing funds for grants or projects [32, 84]. They base this opinion on the altmetrics’ immediate character. However, giving altmetrics a role in assessing a researcher or research group is problematic as any individual researcher or research group can develop multiple strategies to increase the social network dissemination of its publications, and in turn, its Attention Scores: put a link to the article on social networks, post it in blogs, store it in an institutional repository, add the study to professional profiles such as LinkedIn or Google Scholar or send copies of the article by email to colleagues and other authors that are influential in your research field [44]. All this, added to the fact that any social network user can have several different identities means that it is impossible to gauge the real social impact a study or research group is making. For this reason, the idea of distributing funds according to these indicators does not seem reliable at present.

Finally, another major limitation regarding altmetrics is the impossibility of reproducing them and data inconsistency depending on the provider [79, 85]. We do not really know what formula is used to calculate the AAS or Plum Print. Is a tweet from a blog that revises an article worth more than a tweet from a journalist who comments on the article? This type of detail is defined by each provider and is not explained, resulting in a lack of transparency [79, 79]. Given the diversity of sources, collecting all the inputs takes a lot of time and this also contributes to the lack of consistency between providers [84]. Table 4 shows the altmetric data for the article “What the radiologist should know about artificial intelligence—an ESR White paper”, published in *Insights into Imaging.* This data differs depending on whether the AAS or Plum Print is analysed even though both providers were consulted on the same day.

**Table 4:** Altmetric data with different providers

	Total no. of Mendeley reader counts	Twitter interactions	Total no of Facebook interactions	Citations	News
Plum Print	239	136	28	15 (CrossRef citation index)	1
Altmetric score	172	139	4 public wall post (2 users)	38 (publication citations)	2

Article What the radiologist should know about artificial intelligence—an ESR white paper. Date of search: 12 October 2020

The fact that the altmetrics cannot be reproduced is due in part to the publications presenting several different versions (preprints, postprints) and also dependent on which repository it is included in [37], and this can generate ambiguity. To guarantee altmetrics transparency (regarding the way the data aggregators obtain and process the information) [9], the National Information Standards Organization (NISO) developed the NISO Altmetrics Initiative between 2013 and 2016 to establish definitions, calculation methods, improvement in data quality and the use of common identifiers to validate altmetric data [86].

While generally speaking there is a weak relationship between the AAS and the number of citations received, as we saw above, the correlation increases as the publication year becomes more recent. This means that there is a stronger relationship between the AAS and the number of citations received for the most recent articles [55–60, 82]. A recently accepted article has a digital identifier immediately assigned. Editors can use this to know which articles have gained the most social attention and include them in the year’s earlier issues, thus generating a greater number of citations for its IF [42].

## Conclusion

Altmetrics measure the digital attention received by an article using multiple online sources. Due to the fact that data is harvested from a large number of sources and that there is discrepancy between providers, altmetric data should be interpreted with caution. However, it is a constantly evolving and expanding concept with ever increasing correlation between the AAS, article citations and IF. The social impact generated by an article can be useful for discovering which articles are the most popular at any given moment and can even help the journal editor know which articles may go on to be the ones that receive the most citations. However, altmetrics cannot be used to assess new researchers or distribute funds because the way they are calculated is unknown, they are susceptible to manipulation and there are high levels of inconsistency between providers. For all these reasons, altmetrics are not an alternative to the traditional BI, nor will they substitute them. Perhaps in the future, if the methodological limitations of altmetrics are resolved, all facets of a researcher could be evaluated by combining both parameters.

## Data Availability

The structure of the article is “narrative review” (it has not data analysis). This item is not applicable.

## Abbreviations

AAS: Altmetric Attention Score
BI: Bibliometric indicators
IF: Impact factor
